# Alleged Discharge of Hairs from the Hand

**Published:** 1841-12

**Authors:** 


THE WESTERN JOURNAL

Vol. IV.—No. VI.

LOUISVILLE, DECEMBER 1, 1841.

### ALLEGED DISCHARGE OF HAIRS FROM THE HAND.

Our colleague, Prof. Yandell, has received from Dr. Parker T. Shuff, of Scott county, Ky., a letter, dated Nov. 16, 1841, from which we make the following extract:

"Miss Penelope Stout, aged 13, a sprightly girl in good health, about five weeks ago, complained to her mother that the end of her right thumb felt as her foot did when asleep, (such was her own expression;) soon after which, one of the enclosed hairs (if such they can be called) issued from it, and was soon followed by others. Indeed, they have been protruded to the number of from two to ten or twelve, in the 24 hours ever since. The first came out of the ball of the thumb, the subsequent from under the nail. They leave no perceptible aperture. Their escape after they show themselves is almost instantaneous. Some of them are eight or ten inches long. They are of different colors, black, red, and nearly white. You will see that some of the ends are crooked; which is observed to retard their exit."

Since the receipt of this letter, we have conversed with two highly respectable, non-professional gentlemen, who testified to the extraordinary account given by Dr. Shuff. They had seen the hairs issue precisely in the manner just described.

We know not what to say of this *lusus naturæ*, if, indeed, we are to regard it as a reality. Dr. Cullen, the celebrated Edinburg Professor, who was fond of referring to the ancients for explanations of rare phenomena, once on a time gave a supper, at which boiled eggs were served up. A *savant* on opening one of them found in it a large black hair. It was shown to the whole party, when the distinguished professor immediately began to quote the ancients in explanation of the curious phenomenon; in the midst of which, his son announced, that he had perforated the shell and introduced a horse hair before the egg was boiled!

We may conjecture, that the learned professor argued, that as hair had often been found in an *ovarium*, it might be developed in an *ovum*. But hairs are found in various abnormal situations. First on the skin in places where they do not naturally appear. Second, in the mucous membranes. Third, in the ovaries. Fourth, in cysts, or encysted tumors, situated in the subcutaneous cellular tissue, where they are generally embedded in a fatty matter. If a wen were to suppurate, and hairs to issue with the discharge, there would not be any thing wonderful in the phenomenon. But the case before us does not seem to be of this kind. Still, as hairs are developed in cysts, imbeded in the adipose substance, it must be admitted to be possible, that they may be produced in that substance without a cyst; and as aculeated bodies make their way from one part of the system to another, without exciting pain, and finally issue from the surface; it must be granted, that hairs may do the same. But the alleged suddenness of their exit in this case, and the absence of inflammation in the skin, present difficulties which we shall not attempt to surmount, till we are *compelled*, by additional testimony, (not meaning any disrespect to our correspondent,) to believe in the reality of what he reports. Hairs, like pins and needles, when swallowed, might make their way to the surface. Some years since, Dr. Coxe reported in his Philadelphia Medical Museum, a case, in which a head of rye made its way from the stomach of a child, through the parieties of the abdomen; and in the pathological museum of the Medical Institute of Louisville, there is a pin which issued from the surface of the body, many years after it was swallowed.

The hairs, sent us, are of two kinds, black and white, inclining (some of them at least) to amber colour. The former are without bulbs or points, and look like sections. They are coarser than the latter, which, again, are of different lengths and diameters, and have distinct bulbs. Both kind are strong and elastic; burn with an animal odour; sink in water; and, under maceration, become more flexible. Examined with a microscope, they exhibit the usual reticulated structure.

The date of Dr. Shuff's letter is the 16th of November, five weeks, as he states, after the first hair appeared. Ten days subsequently, one of our informants saw them issuing.

Such are the statements which have reached us. If they are a fabrication, why then, gentle reader! you have lost as much time in reading as we have in writing, *et vice versa,* and so the account between us is balanced. But you may say, that if we have been hoaxed, it is no excuse for hoaxing you; to which we reply, that you need not be hoaxed unless you choose; that public journalists are always liable to be hoaxed; and that it is a less calamity to be made the subject of a joke, than to be always guarding against it. A hoax moreover is a harmless thing; in general leading to nothing more than a hearty laugh, which depurates the blood and promotes health and cheerfulness. In our next number we may perhaps give you a warrant for such a laugh.

